# Estimation of the ion-trap assisted electrical loads and resulting BBR shift

**DOI:** 10.1038/s41598-018-35234-5

**Published:** 2018-11-15

**Authors:** Lakhi Sharma, A. Roy, S. Panja, V. N. Ojha, S. De

**Affiliations:** 1 CSIR-National Physical Laboratory, Dr. K. S. Krishnan Marg, New Delhi, 110012 India; 2 Academy of Scientific and Innovative Research (AcSIR), Ghaziabad, 201002 India

## Abstract

Capacitive, inductive and resistive loads of an ion-trap system, which can be modelled as LCR circuits, are important to know for building a high accuracy experiment. Accurate estimation of these loads is necessary for delivering the desired radio frequency (RF) signal to an ion trap via an RF resonator. Of particular relevance to the trapped ion optical atomic clock, determination of these loads lead to accurate evaluation of the Black-Body Radiation (BBR) shift resulting from the inaccurate machining of the ion-trap itself. We have identified different sources of these loads and estimated their values using analytical and finite element analysis methods, which are found to be well in agreement with the experimentally measured values. For our trap geometry, we obtained values of the effective inductive, capacitive and resistive loads as: 3.1 *μ*H, 3.71 (1) *μ*H, 3.68 (6) *μ*H; 50.4 pF, 51.4 (7) pF, 40.7 (2) pF; and 1.373 Ω, 1.273 (3) Ω, 1.183 (9) Ω by using analytical, numerical and experimental methods, respectively. The BBR shift induced by the excess capacitive load arising due to machining inaccuracy in the RF carrying parts has been accurately estimated, which results to a fractional frequency shift of 6.6 × 10^−17^ for an RF of 1 kV at 2*π* × 15 MHz and with ±10 *μ*m machining inaccuracy. This needs to be incorporated into the total systematic uncertainty budget of a frequency standard as it is about one order of magnitude higher than the present precision of the trapped ion optical clocks.

## Introduction

An ion-trap system either for guiding^1,2^ or for trapping^3,4^ of ions requires narrow band radio frequency (RF) at the desired parameters. It is equivalent to an LCR-circuit having different impedance than the 50 Ω output impedance of an RF source. Using a directional device following an RF source is a standard technique to avoid unwanted power reflection due to impedance mismatching. So, the RF is generally delivered via inductive coupling through an intermediate resonator whose resonant condition is tuned to obtain the desired RF parameters. Over the last few decades, increasing application of the ion-traps for mass spectrometry, precision measurements, generating ion-qubit for quantum computation, development of optical clocks, *etc*. has been demanding high quality RF oscillator and resonator together with improvement in all the associated technologies to achieve an overall high performance.

Focussing on the objectives of this article, here, we briefly review the relevant work on the RF resonator and oscillator. Jones *et al*. reported novel design of an RF generator that allows for tuning of the resonant frequency which varies with the external capacitive loads^5,6^. Recently, Reza *et al*. reported a tunable Colpitts oscillator that also tunes the RF at a desired frequency^7^. In many cases, the resonant frequency of an RF generator is adjusted by adding extra capacitor in parallel to the ion-trap’s intrinsic capacitance but that degrades the quality factor (*Q*-factor) and efficiency of the power transferred to the trap electrodes. As reported in ref.^8^, the operating frequency of their indigenously developed RF amplifier got shifted by about 13.6% than it was designed for, which is due to the lack of prior knowledge on the load of their ion-trap. In all these above-mentioned cases, prior analysis and accurate estimation of the electrical loads will help in proper designing of the RF oscillator. In some applications, feedback controlled RF oscillators are used to enhance the stability^9^. There, a Phase-Lock-Loop locks the resonant frequency, which depends on the extra phase added to the RF due to the propagation delay^10^. Estimation of the loads that are attached to the RF generator and their electrical equivalent circuitry analysis helps in proper designing of the feedback system. For ion ejections, fast discharge of the RF after switching it off is required, otherwise the ions are deflected due to residual oscillations^11^. As the characteristic discharge time of a RF source depends on the effective inductive and capacitive loads^12^, estimation of these is required prior to designing of the RF supply.

So far, efforts have been made for novel designing of the RF generator and oscillator for developing high performance ion-traps. In this article, we show a pathway for accurate estimation of the capacitive, inductive and resistive loads resulting from an ion-trap and its associated system. Towards designing of a precision ion trap that will be used for building of a Yb-ion optical clock^13,14^, we have estimated electrical equivalent loads resulting from different sources and verified them experimentally. This helps us to build a desired helical resonator^15,16^ and to estimate the BBR shift resulting from resistive heating of the ion-trap.

## Methodology

The equivalent inductances, *L*_*i*_; capacitances, *C*_*i*_ and resistances, *R*_*i*_ resulting from various components, as indicated by the subscript (*i*), of an ion-trap appear as loads. Due to impedance mismatch, direct connection of the RF source to an ion-trap not only leads to back reflection but also injects intrinsic noise of the RF source to the confining potential, which results to instability of the ions trapped in it. To overcome these, a helical coil resonator is generally used for applying the RF to the ion-trap^17,18^. This also acts as a bandpass filter and suppresses the noise. The resonant frequency, *f*_0_ and *Q*-factor, *Q*_0_ of a helical resonator itself can be estimated as,

1 $${f}_{0}=\frac{1}{2\pi }\sqrt{\frac{1}{{L}_{R}{C}_{R}}},$$

2 $${Q}_{0}=\frac{1}{{R}_{R}}\sqrt{\frac{{L}_{R}}{{C}_{R}}},$$

where *L*_*R*_, *C*_*R*_ and *R*_*R*_ are the inductance, capacitance and resistance of the resonator, respectively^18^. In a loaded condition, *i*.*e*., when the ion-trap is attached to the resonator via a connector, the *f*_0_ and *Q*_0_ changes as,

3 $$f^{\prime} =\frac{1}{2\pi }\sqrt{\frac{1}{({L}_{R}+{L}_{L})({C}_{R}+{C}_{L})}},$$

4 $$Q^{\prime} =\frac{1}{{R}_{R}+{R}_{L}}\sqrt{\frac{{L}_{R}+{L}_{L}}{{C}_{R}+{C}_{L}}},$$

where *L*_*L*_, *C*_*L*_ and *R*_*L*_ are the inductive, capacitive and resistive loads, respectively. The ratio of the effective RF voltage, *V*′ at the trap electrode relative to the input voltage, *V* is given as,

5 $${k}_{RF}=\frac{V^{\prime} }{V}=\sqrt{\frac{{R}_{R}{C}_{R}}{({R}_{R}+{R}_{L}\mathrm{)\ (}{C}_{R}+{C}_{L})}}\mathrm{.}$$

**Figure 1:**
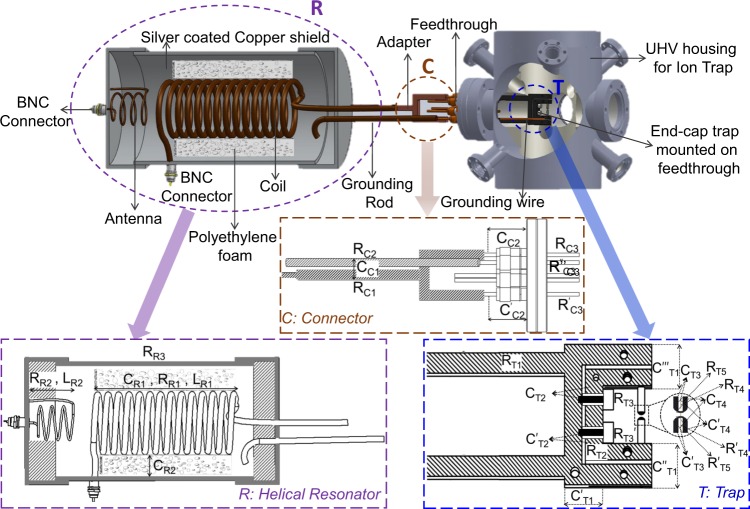
Ion-trap system of our experiment where the resonator (**R**) and ion-trap (**T**) are connected via a connector (**C**). The inserts show schematics of each part **R**, **C** and **T** where sources of the loads are indicated.

Our ion-trap system, which is attached to an amplified RF source, consists of three parts: a helical resonator **R**, a connector **C** and the end cap type Paul trap **T**. Their arrangements are shown in Fig. 1. Here, we have identified as well as estimated possible sources of loads that contribute to *L*_*L*_, *C*_*L*_ and *R*_*L*_ which are then validated through experiment.

### Helical Resonator (R)

**Figure 2:**
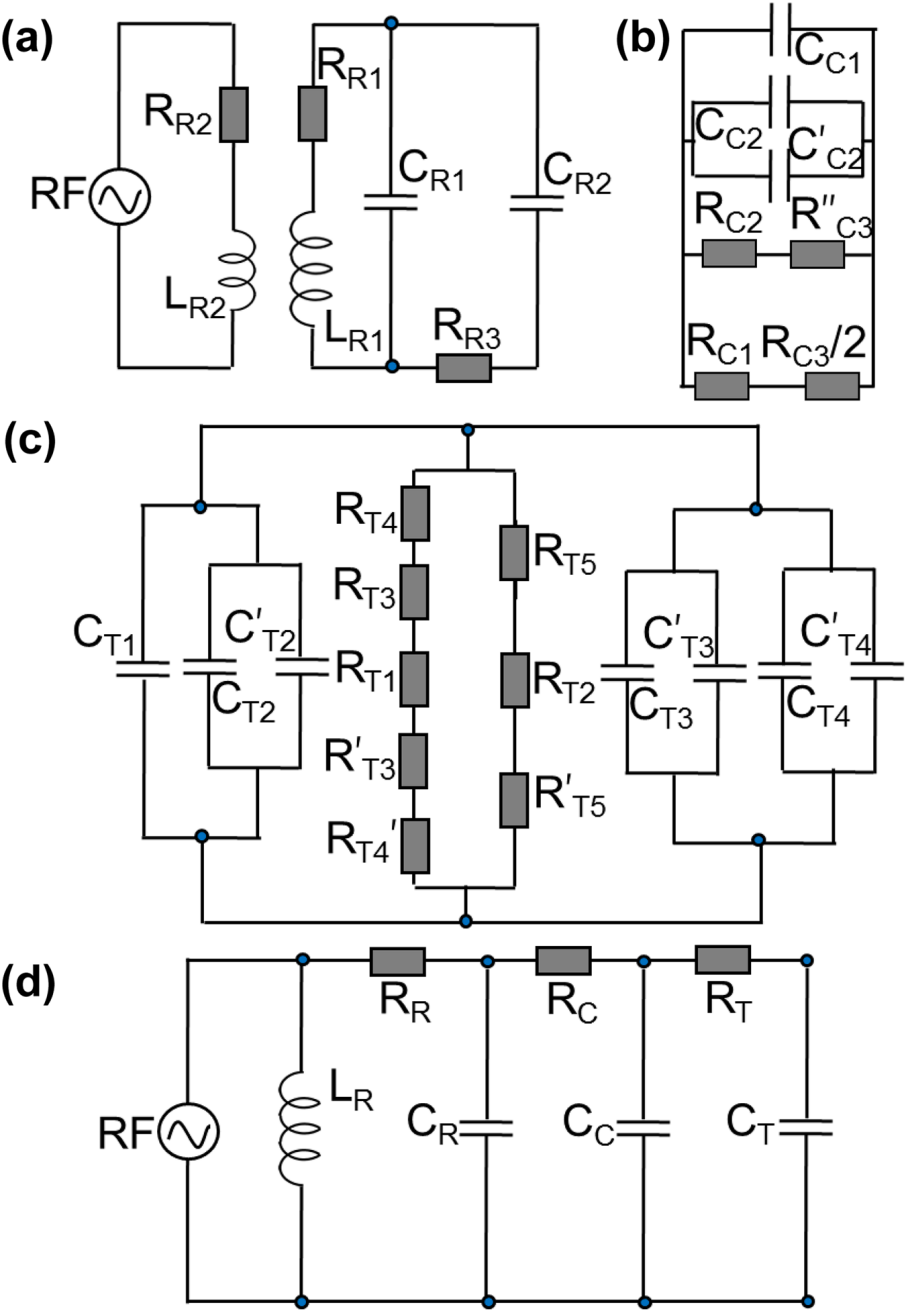
Electrical circuitry of (**a**) resonator (**R**), (**b**) connector (**C**), (**c**) ion trap (**T**) and (**d**) equivalent (**E**) of all three components. *C*_*i*_, *R*_*i*_ and *L*_*i*_ as shown in the circuits denote capacitance, resistance and inductance, respectively, where the subscript *i* refers to **R**, **C** and **T**, respectively.

The design parameters of our helical resonator are described in the earlier publications^15,16^. The antenna and the secondary coil have self inductances, *L*_*R*1_ and *L*_*R*2_; resistances, *R*_*R*1_ and *R*_*R*2_ and a parasitic capacitance, *C*_*R*1_. Capacitance *C*_*R*2_ results from the shield-air-coil combination. In the present design, the copper enclosure is silver coated to reduce the electromagnetic radiation loss, which introduces a resistance *R*_*R*3_. Electrical circuitry comprising of these loads are shown in Fig. 2(a). For calculation of the self capacitance of a helical coil of height *h*_*c*_ and outer diameter *d*_*c*_, the modified Medhurst equation^19^ as given by D. W. Knight^20^ is,

6 $${C}_{coil}=\frac{4{\varepsilon }_{0}{\varepsilon }_{x}}{\pi }\,\mathrm{[1}+\frac{{k}_{c}}{2}\,\mathrm{(1}+\frac{{\varepsilon }_{i}}{{\varepsilon }_{x}})]\,\frac{{h}_{c}}{{cos}^{2}{\rm{\Psi }}},$$

where *ε*_0_, *ε*_*i*_ and *ε*_*x*_ are permittivities of free space, inside and outside mediums of the coil, respectively and Ψ is the pitch angle. The coefficient *k*_*c*_ is expressed as,

7 $${k}_{c}=0.717439\frac{{d}_{c}}{{h}_{c}}+0.933048{(\frac{{d}_{c}}{{h}_{c}})}^{\frac{3}{2}}+0.106\,{(\frac{{d}_{c}}{{h}_{c}})}^{2},$$

where the numerical pre-factors are the empirical coefficients^20^. Assuming the shield and the coil as coaxial cylinders, the shield-to-coil (s-c) capacitance is calculated using the relation,

8 $${C}_{s-c}=2\pi {\varepsilon }_{o}{\varepsilon }_{r}{h}_{c}{(ln\frac{{d}_{s}}{{d}_{c}})}^{-1},$$

where *d*_*s*_ is the inner diameter of the shield and *ε*_*r*_ is the relative permittivity of any dielectric material in between. For proper positioning of the coil, the space between the shield and coil is partially filled with polyethylene foam of permittivity 2.22. Hence, following the Eqns (6–8), the capacitances of the coil and shield-to-coil are,

9 $${C}_{coil}^{^{\prime} }=m\,{C}_{coil}{|}_{{\varepsilon }_{i},{\varepsilon }_{x}=1}+n\,{C}_{coil}{|}_{{\varepsilon }_{i}=1,{\varepsilon }_{x}=2.22},$$

10 $${C}_{s-c}^{^{\prime} }=m\,{C}_{s-c}{|}_{{\varepsilon }_{r}=1}+n\,{C}_{s-c}{|}_{{\varepsilon }_{r}=2.22},$$

here *m* = 0.46 and *n* = 0.54 are the filling factors for air and foam, respectively. Inductance of the coil is estimated as,

11 $${L}_{coil}=39.37\,\frac{{\mu }_{0}}{16\pi }\,\frac{{h}_{c}\,{d}_{c}^{2}}{{\tau }^{2}}[1-{(\frac{{d}_{c}}{{d}_{s}})}^{2}],$$

where *τ* is the winding pitch of the coil and the numerical term arises from unit conversion of the formula, as given in ref.^21^ to the SI. The DC resistance of a part of length *l* and cross-sectional area *A* is given by,

12 $${R}_{dc}=\frac{\rho l}{A},$$

where *ρ* is the resistivity of the material. AC resistances for the RF carrying parts are different and those are important to be calculated. For a cylindrical rod of diameter *D*, the AC resistance^22^ is,

13 $${R}_{ac}^{c}=\frac{{R}_{dc}}{\pi }\,\frac{A}{D\delta },$$

where the skin depth of the material is $\delta =503\,\sqrt{\rho /{\omega }_{RF}\,\mu }$ with *ω*_*RF*_ and *μ* being the applied frequency and the relative permeability of the material, respectively. *R*_*R*1_ and *R*_*R*2_ are calculated using Eqns (12) and (13).

### Connector (C)

A connector is used to interface the helical resonator with the ion-trap, which is assembly of a Y-shaped copper adapter, a grounding rod and an electrical feedthrough (Fig. 1). The Y-shaped adapter is used for symmetric distribution of the RF to the ion-trap electrode pair. The trap is screwed to two radially symmetric electrical pins of the feedthrough for firm and stable mounting. The capacitance, *C*_*C*1_ arises between the adapter and the grounding rod along with the feedthrough pins whereas *C*_*C*2_ and ${C^{\prime} }_{C2}$ results from the feedthrough pins and its flange. The adapter, grounding rod, feedthrough pins also carry resistances *R*_*C*1_, *R*_*C*2_ and *R*_*C*3_, respectively. The rectangular parts of the connector of width *w* and thickness *t* carrying RF has an AC resistance^23^ which is given as,

14 $${R}_{ac}^{r}={R}_{dc}\,\frac{{K}_{c}}{1-{e}^{-x}},$$

where *x* = 2 (1 + *t*/*w*) *δ*/*t*, current crowding factor *K*_*c*_ = 1 + (1 − *e*^−0.048*p*^) [0.06 + 0.22 *ln*(*w*/*t*) + 0.28 (*t*^2^/*w*^2^)] and *p* = $\sqrt{A}/1.26\,\delta$. Equivalent electrical circuitry of all the loads described in this section are shown in Fig. 2(b).

### Trap (T)

The ion-trap electrodes are made of Tantalum, whereas its holding structure is made of Molybdenum and Macor^24^. The holder mounts the ion-trap inside an Ultra High Vacuum (UHV) chamber via an electrical feedthrough. Capacitances in the trap arise out of the inner electrode holder to outer electrode holder with dielectric macor in between. This capacitance, *C*_*T*1_ is estimated part-by-part as *C*′_*T*1_, *C*′′_*T*1_ and *C*′′′_*T*1_ as shown in Fig. 1. The capacitances resulting from the mounting bolts and the electrode holders are *C*_*T*2_ and ${C^{\prime} }_{T2}$, respectively and from the inner-to-outer electrodes are *C*_*T*3_, ${C^{\prime} }_{T2}$, *C*_*T*4_ and ${C^{\prime} }_{T4}$. Resistances result from the inner electrode holder, *R*_*T*1_; outer electrode holder, *R*_*T*2_; bolts, *R*_*T*3_; inner and outer electrode, *R*_*T*4_, *R*_*T*5_ and ${R^{\prime} }_{T4}$ and ${R^{\prime} }_{T5}$, respectively. Trap’s inner and outer electrode holders can be considered as parallel plates spaced apart by macor of thickness *d*. In that case, the capacitances are calculated as,

15 $$C={\varepsilon }_{0}{\varepsilon }_{r}\frac{A}{d}.$$

Since, the trap electrodes are cylindrical in shape, their in-between capacitance is calculated from Eqn. (8) by replacing *h*_*c*_, *d*_*c*_ and *d*_*s*_ with the length, inner and outer diameters of the outer and inner electrodes, respectively. Equation (14) gives *R*_*T*1_ whereas Eqn. (13) is utilized for calculating *R*_*T*3_ and *R*_*T*4_. The electrical equivalent circuitry of the loads associated to the ion-trap is shown in Fig. 2(c).

### Numerical and Analytical methods

We perform finite element analysis via COMSOL Multiphysics software (COMSOL) for numerical estimation of various *L*_*i*_, *C*_*i*_ and *R*_*i*_ values. The geometric designs of the parts are fed into the COMSOL and subdivided into finite size elements as per the user’s choice. The software analyzes them and generates a final solution using variational method. The computational time increases dramatically with finer mesh size, however, after a certain mesh size, the incremental change of the computed value is well within the limit of our desired accuracy. We use the AC/DC electromagnetics module of COMSOL as the subnodes: electrostatics interface, electric current and magnetostatics allow direct computation of capacitances, resistances and inductances, respectively.

For analytical estimation, we use the formulae as described in the Sec. III A-C, which rely on certain assumptions. As for example, *C*_*i*_ values are estimated assuming parallel plate capacitors. Thus, we do not expect them to be as accurate as for the numerical techniques but this shows inaccuracy of the estimation in case it is done only analytically.

### Experiment

Experimental verification of the estimated values are obtained through an indirect measurement as well as via a direct measurement of the loads using an LCR meter. For indirect measurement, the resonant condition is obtained by scanning the RF frequency across *f*_0_ and *f*′ as given in Eqns (1 and 3). A small fraction of the RF (≤3%) gets reflected from the resonator. Using a directional coupler, this reflected signal is outcoupled to a spectrum analyzer for further analysis. The resonance condition is ensured by minimum reflection of the RF while tuning its frequency. The outcoupled RF is measured in a spectrum analyzer at different values of fixed capacitive loads and also in the unloaded condition of the resonator.

## Results and Discussions

Simple circuitry analysis using Kirchoff’s current law leads to estimation of the effective loads as following, for the Resonator **R**:

16 $$\begin{array}{rcl}{C}_{R} & = & {C}_{R1}+{C}_{R2},\\ {R}_{R} & = & {R}_{R1}+{R}_{R2}+{R}_{R3},\\ {L}_{R} & = & \frac{{L}_{R1}{L}_{R2}}{{L}_{R1}+{L}_{R2}},\end{array}$$

for the Connector **C**:

17 $$\begin{array}{rcl}{C}_{C} & = & {C}_{C1}+{C}_{C2},\\ {R}_{C} & = & \frac{\mathrm{(2}{R}_{C1}+{R}_{C3}\mathrm{)\ (}{R}_{C2}+{R}_{C3})}{\mathrm{2(}{R}_{C1}+{R}_{C2})+3{R}_{C3}},\end{array}$$

for the Trap **T**:

18 $$\begin{array}{rcl}{C}_{T} & = & {C}_{T1}+\mathrm{2(}{C}_{T2}+{C}_{T3}+{C}_{T4}),\\ {R}_{T} & = & \frac{AB}{A+B},\end{array}$$

where, *C*_*T*1_ = (*C*_*T*1_′ + *C*_*T*1_′′ + *C*_*T*1_′′′) as indicated in Fig. 1, *A* = (*R*_*T*2_ + 2*R*_*T*5_) and *B* = (*R*_*T*1_ + 2*R*_*T*3_ + 2*R*_*T*4_), for total effective loads **E**:

19 $$\begin{array}{rcl}{C}_{E} & = & {C}_{R}+{C}_{T}+{C}_{C},\\ {R}_{E} & = & {R}_{R}+{R}_{T}+{R}_{C},\\ {L}_{E} & = & {L}_{R}\mathrm{.}\end{array}$$

The wire that is used for grounding of the trap inside of the UHV chamber results in a resistive load. The extra compensation electrodes, that are generally used for precise positioning of the ion at the trap centre, also results to finite capacitance. These values have been estimated to be orders of magnitude lower than the rest, which are therefore neglected.

The values of the loads as given in Eqns (16–18), estimated via analytical and numerical techniques are given in Table 1. Source of errors in the numerically calculated values are two fold: accuracy *a* that is obtained from COMSOL for a certain mesh size and their inaccuracy *b* for ±10 *μ*m machining tolerance. Hence, the total error is estimated to be ±$\sqrt{{a}^{2}+{b}^{2}}$, which is dominated by *b*.

**Table 1:** Estimated values of the loads resulting from different parts of the ion-trap system.

Parts	Analytical	Numerical
**Resonator** (**R**)		
*C*_*R*1_	3.7 pF	2.067 (5) pF
*C*_*R*2_	5.7 pF	10.72 (1) pF
*R*_*R*1_	7 *m*Ω	8 (1) *m*Ω
	351 *m*Ω^*ac*^	450 (5) *m*Ω^*ac*^
*R*_*R*2_	18 *m*Ω	10 (4) *m*Ω
	820 *m*Ω^*ac*^	610 (9) *m*Ω^*ac*^
*R*_*R*3_	5.2 *μ*Ω	5.83 (3) *μ*Ω
*L*_*R*1_	3.1 *μH*	3.71 (6) *μH*
*L*_*R*2_	0.3 *μH*	0.242 (5) *μH*
**Connector (C)**		
*C*_*C*1_	8.2 pF	9.8 (2) pF
*C*_*C*2_	0.04 pF	0.038 (7) pF
*R*_*C*1_	0.22 *m*Ω	0.222 (1) *m*Ω
	6 *m*Ω^*ac*^	7 (1) *m*Ω^*ac*^
*R*_*C*2_	454.55 *μ*Ω	455.8 (4) *μ*Ω
${R}_{C3},R{^{\prime} }_{C3}$	347.6 *μ*Ω	348.35 (3) *μ*Ω
	10 *m*Ω^*ac*^	9 (2) *m*Ω^*ac*^
**Trap (T)**		
*C*_*T*1_	22.5 pF	22.1 (3) pF
${C}_{T2},{C}_{T2}^{^{\prime} }$	0.8 pF	0.71 (2) pF
${C}_{T3},{C}_{T3}^{^{\prime} }$	1.6 pF	1.5 (1) pF
${C}_{T4},{C}_{T4}^{^{\prime} }$	0 pF	0.042 (4) pF
*R*_*T*1_	0.073 *m*Ω	0.078 (1) *m*Ω
	70 *m*Ω^*ac*^	85 (3) *m*Ω^*ac*^
*R*_*T*2_	80.1 *μ*Ω	81.1 (3) *μ*Ω
${R}_{T3},{R^{\prime} }_{T3}$	189.44 *μ*Ω	197.8 (3) *μ*Ω
	3.2 *m*Ω^*ac*^	3.4 (2) *m*Ω^*ac*^
${R}_{T4},{R^{\prime} }_{T4}$	2.816 *m*Ω	2.790 (2) *m*Ω
	15 *m*Ω^*ac*^	14 (1) *m*Ω^*ac*^
${R}_{T5},{R^{\prime} }_{T5}$	780.3 *μ*Ω	788.6 (1) *μ*Ω

AC resistances are indicated by superscript ‘ac’.

**Figure 3:**
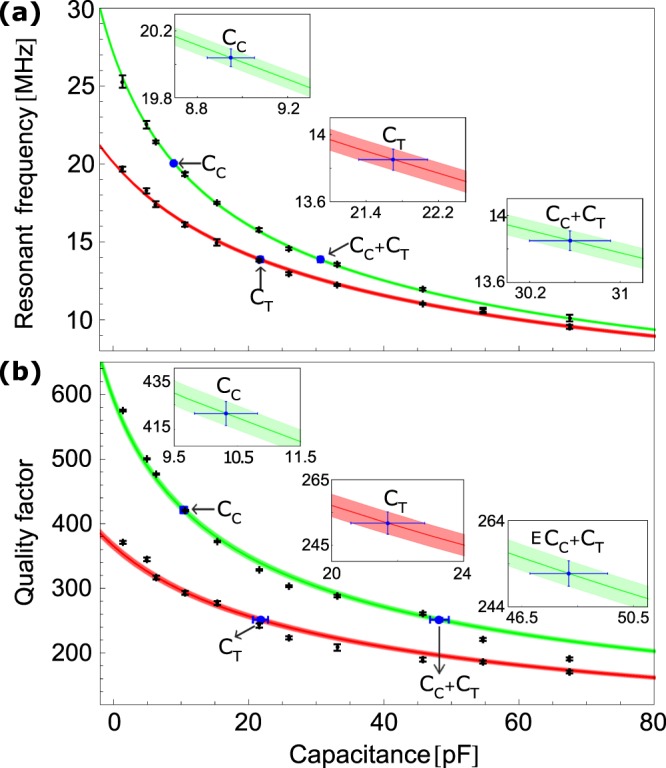
Variation of the (**a**) resonant frequency and (**b**) quality factor with capacitance for the resonator itself (green) and for the resonator + connector without the ion-trap connected to it (red). The experimental data (black) and theoretically fitted lines to it are shown, where the width of the lines depict fitting inaccuracy. The measured capacitance (blue) of connector and the combination of connector and trap are indicated on the fitted curves and also shown in the inserts.

In the experiment, we measure resonant frequencies and *Q*-factors when: (i) the resonator is attached to capacitors and (ii) the resonator is attached to the connector and capacitors. For this purpose, we use capacitors with different values that are calibrated at 0.3% uncertainty. As shown in Fig. 3, the characteristic change of the resonant frequency and *Q*-factor due to additional capacitive load are fitted to $f({C}_{L})={f}_{0}\,\sqrt{{C}_{R}/({C}_{L}+{C}_{R})}$ and $Q({C}_{L})={Q}_{0}\,\sqrt{{C}_{R}/({C}_{L}+{C}_{R})}$, respectively, where *f*_0_, *Q*_0_ and *C*_*R*_ are used as the fit parameters. Capacitive load resulting from a component attached to the resonator is obtained by measuring shift of the resonant frequency or *Q*-factor from the unloaded *f*_0_, *Q*_0_ values and obtaining change of capacitance corresponding to that shift from their respective characteristic curves, as given in Fig. 3. With the known values of *f*_0_ and *f*′ for any attached capacitance *C*_*i*_, its unknown value of *L*_*i*_ can be estimated by using Eqns (1) and (3). Further, as the *Q*_0_ and *Q*′ values are also known from experiments, using the estimated *L*_*i*_ values, the unknown *R*_*i*_ values can be obtained using the Eqns (2) and (4). The experimental values are obtained by taking mean of the values taken from different techniques and their variance is quoted as the uncertainty. We measure *f*_0_ = 27.04 (7) MHz and *Q*_0_ = 600 (8), estimate them as 25.85 (5) MHz and 508 (1) using the numerically estimated loads in Eqns (1–2) and also extract them as 27.5 (1.0) MHz and 590 (7) from the fits. Mutual agreement of these values also validates our calculation in an independent way. The analytical, numerical and experimental values of different loads are compared in Table 2. Using the numerical and experimental values of the loads in Eqn. (5), we estimate the RF voltage transfer factor, *k*_*RF*_ ≃ 0.45 and 0.46, respectively. Therefore, voltage at the trap is about half of that is at the output of RF resonator.

**Table 2:** Values of the loads obtained through different methods.

Parts	Analytical	Numerical	Experimental
*C*_*R*_	9.4 pF	12.78 (2) pF	10.6 (1) pF
*C*_*C*_	8.24 pF	9.9 (2) pF	8.6 (4) pF
*C*_*T*_	27.3 pF	26.7 (5) pF	21.5 (9) pF
*R*_*R*_	1241 *m*Ω	1062 (1) *m*Ω	981 (5) *m*Ω
*R*_*C*_	28 *m*Ω	29 (9) *m*Ω	44 (2) *m*Ω
*R*_*T*_	174 *m*Ω	182 (3)*m*Ω	162 (2) *m*Ω
*L*_*R*_	3.1 *μH*	3.71 (1) *μH*	3.68 (6) *μH*
***C***_***E***_	**50.4** ***pF***	**51.4 (7)** ***pF***	**40.7 (2)** ***pF***
***R***_***E***_	**1373** ***m*Ω**	**1273 (3)** ***m*Ω**	**1183 (9)** ***m***Ω
***L***_***E***_	**3.1** ***μH***	**3.71 (1) ** ***μH***	**3.68 (6) ** ***μH***

**Figure 4:**
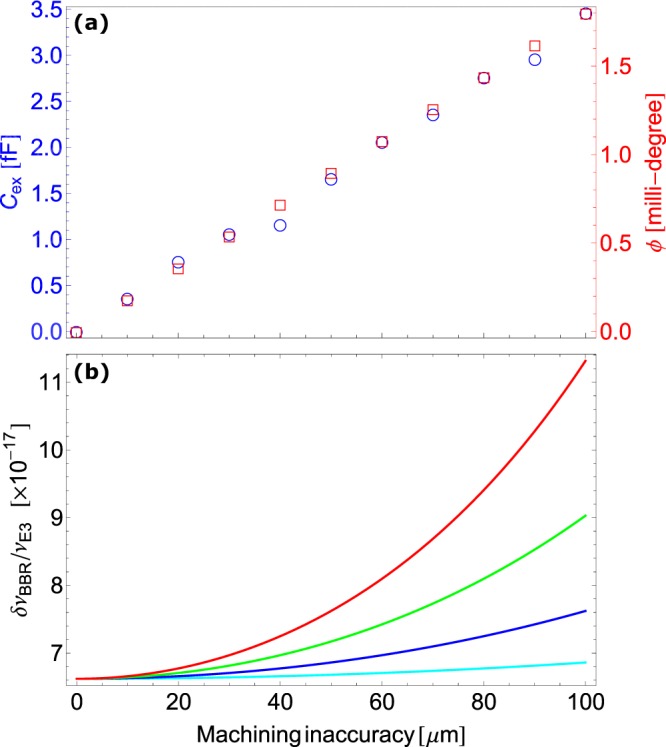
(**a**) Excess capacitance, *C*_*ex*_ and phase difference, *ϕ* and (**b**) the BBR shift, at RF amplitudes 250 V (cyan), 500 V (blue), 750 V (green) and 1000 V (red) at the trap electrodes; resulting from the machining inaccuracy are shown.

The described electrical load analysis also leads to estimation of the excess BBR shift that results from heating of the ion-trap. The resistive heating of the ion-trap electrodes due to absorption of the RF power propagating through it has already been reported elsewhere^25–27^. Here, we are estimating the BBR shift associated to heating of the trap due to current flowing through it, which originates due to finite capacitance introduced by inaccurate machining of the trap parts. The RF appears at a different phase with a relative phase difference *ϕ* on the trap electrodes, that are aligned axially facing each other, due to a path difference *x* resulting from non-negligible machining tolerances. Due to this, at any instant of time, the RF, *V*_*o*_*sinω*_*RF*_*t*, appears at different voltage level, which can be modelled as a parallel plate capacitor with an unwanted excess capacitance *C*_*ex*_ kept at a potential *V*_*o*_*ϕ*^28^. The phase difference, resulting from the inaccurate machining, is

20 $$\varphi =2\pi (\frac{x}{{\lambda }_{RF}}-p),$$

where *p* is the integer number of RF wavelength *λ*_*RF*_ that gets accommodated within *x*. Figure 4(a) shows numerically estimated values of *C*_*ex*_ for our ion-trap and calculated *ϕ* following the Eqn. (20) as a function of the machining inaccuracy. Even though there is no direct relation, the capacity factor in our ion-trap geometry is *α*_*c*_ = *C*_*ex*_/*ϕ* = 1.9, where *C*_*ex*_ and *ϕ* are in fF and milli-degree, respectively. Although, in an ideal condition (*ϕ* = 0), the RF electrodes stay in an open circuit condition, a finite current *I*_*ex*_ = *V*_*o*_*ϕ*/*R*_*RF*_ flows through in a real trap that has certain capacity factor. A simple minded physical picture to understand this is by considering a voltage source of *V*_*o*_*ϕ* V that is present in between the electrodes. In that case, the resulting *I*_*ex*_ flows through the resistance *R*_*RF*_ that is present in the RF path of the ion-trap system. In a thermal equilibrium, the resistive heating due to *I*_*ex*_ will elevate temperature of the trap, which is non-negligible in many precision experiments particularly in case of an optical clock as that significantly contributes to the BBR shift^29^. The amount of temperature rise during the time of current flow (*i*.*e*. cycle time of the experiment and we have considered that to be 1 s just for simplicity) can be estimated following the conservation of energy as

21 $$\begin{array}{rcl}{\rm{\Delta }}T & = & \frac{{V}_{o}^{2}{\varphi }^{2}}{{R}_{RF}\sum _{j}\,{m}_{j}{s}_{j}}\\  & = & \frac{{C}_{ex}^{2}{V}_{o}^{2}}{{\alpha }_{c}^{2}{R}_{RF}\sum _{j}\,{m}_{j}{s}_{j}},\end{array}$$

where *m*_*j*_ and *s*_*j*_ are masses and specific heats of the elements *j*, respectively. Corresponding BBR shift due to the electromagnetic radiation at an elevated temperature *T*^30^ can be estimated as,

22 $${\rm{\Delta }}{\nu }^{BBR}=-\frac{{\mathrm{(831.945)}}^{2}}{2h}\delta {\alpha }_{o}{(\frac{T}{300})}^{4}\,\mathrm{[1}+\eta (T)],$$

where *h* is the Planck’s constant, *δα*_*o*_ is differential scalar polarizability of the transition and *η*(*T*) is the dynamic correction since atomic transitions other than the E1-transitions are neglected in *δα*_*o*_. Here, we have neglected this factor since *η* < 0.01 at room temperature. As an example, here we estimate Δ*ν*^*BBR*^ at T = (296 + ΔT) that is from the machining inaccuracy for the Ytterbium-ion (^171^Yb^+^) octupole (E3) clock transition at frequency *ν*_*E*3_ = 642121496772645.150 Hz^31^ and having *δα*_*o*_ = 0.859 × 10^−40^ Jm^2^V^−2^ ^32^. We use our estimated values of resistances, as given in Table 1, to get ${R}_{RF}=({R}_{T1}+{R}_{T3}+{R^{\prime} }_{T3}+{R}_{T4}+{R^{\prime} }_{T4}+{R}_{C1}+{R}_{C3}+{R^{\prime} }_{C3})$. Figure 4(b) shows expected fractional BBR-shift Δ*ν*^*BBR*^/*ν*_*E*3_ resulting from the machining inaccuracy of the electrodes at different amplitudes of RF of *ω*_*RF*_ = 2*π* × 15 MHz. Accurate estimation of this systematic shift carries merit for developing optical clocks with increasing accuracies as it is about two orders of magnitude higher than the present best trapped ion optical frequency standard^31^.

## Conclusion

We made detailed analysis of the resistive, inductive and capacitive loads that results from each part of the ion-trap system. We also performed an experiment to measure the effective loads to validate our theoretical estimations. Considering the estimated load values, the design parameters of the resonator can be chosen such that it operates as desired. This analysis shows a pathway for predicting output values, *e*.*g*., resonant frequency, *Q*-factor of an RF resonator both in loaded and unloaded conditions prior to its construction. The RF phase difference at the tip of the electrodes resulting from the unignorable machining inaccuracy, has been estimated accurately. Its relation with one of the dominant systematic shifts in the atomic clock experiments: BBR shift is obtained, which is important to be considered in the overall systematic budget. As for example, at an RF amplitude of 1 kV at 2 *π* × 15 MHz and with ±10 *μ*m machining inaccuracy will result to ±0.42 K heating of the trap. A temperature increase (decrease) will result to -43 (+43) mHz shift for the Yb ion E3-clock transition that corresponds to fractional frequency uncertainty 6.6 × 10^−17^ due to the BBR effect. This exercise for precise estimation of the machining inaccuracy assisted BBR shift helps in building highly accurate optical frequency standards that are approaching to 10^−18^ level even without any cryogenic environment.
